# Laterally extended atomically precise graphene nanoribbons with improved electrical conductivity for efficient gas sensing

**DOI:** 10.1038/s41467-017-00692-4

**Published:** 2017-10-10

**Authors:** Mohammad Mehdi Pour, Andrey Lashkov, Adrian Radocea, Ximeng Liu, Tao Sun, Alexey Lipatov, Rafal A. Korlacki, Mikhail Shekhirev, Narayana R. Aluru, Joseph W. Lyding, Victor Sysoev, Alexander Sinitskii

**Affiliations:** 1 Department of Chemistry, University of Nebraska-Lincoln, Lincoln, Nebraska 68588 USA; 2 Department of Physics, Gagarin State Technical University of Saratov, Saratov, 410054 Russia; 3 Beckman Institute for Advanced Science and Technology, University of Illinois at Urbana-Champaign, Urbana, Illinois 61801 USA; 4 Department of Materials Science and Engineering, University of Illinois at Urbana-Champaign, Urbana, Illinois 61801 USA; 5 Department of Electrical and Computer Engineering, University of Illinois at Urbana-Champaign, Urbana, Illinois 61801 USA; 6 Department of Mechanical Science and Engineering, University of Illinois at Urbana-Champaign, Urbana, Illinois 61801 USA; 7 Department of Electrical and Computer Engineering, University of Nebraska–Lincoln, Lincoln, Nebraska 68588 USA; 8 National University of Science and Technology MISIS, Moscow, 119991 Russia; 9 Nebraska Center for Materials and Nanoscience, University of Nebraska-Lincoln, Lincoln, Nebraska 68588 USA

## Abstract

Narrow atomically precise graphene nanoribbons hold great promise for electronic and optoelectronic applications, but the previously demonstrated nanoribbon-based devices typically suffer from low currents and mobilities. In this study, we explored the idea of lateral extension of graphene nanoribbons for improving their electrical conductivity. We started with a conventional chevron graphene nanoribbon, and designed its laterally extended variant. We synthesized these new graphene nanoribbons in solution and found that the lateral extension results in decrease of their electronic bandgap and improvement in the electrical conductivity of nanoribbon-based thin films. These films were employed in gas sensors and an electronic nose system, which showed improved responsivities to low molecular weight alcohols compared to similar sensors based on benchmark graphitic materials, such as graphene and reduced graphene oxide, and a reliable analyte recognition. This study shows the methodology for designing new atomically precise graphene nanoribbons with improved properties, their bottom-up synthesis, characterization, processing and implementation in electronic devices.

## Abstract (web summary)

Atomically precise graphene nanoribbons are a promising platform for tailored electron transport, yet they suffer from low conductivity. Here, the authors devise a strategy to laterally extend conventional chevron nanoribbons, thus achieving increased electrical conductivity and improved chemical sensing capabilities.

## Introduction

Atomically precise graphene nanoribbons (GNRs) have received a great deal of attention from researchers because of their promising electronic properties and potential applications in electronics, photovoltaics, sensors, and spintronics^1–4^. However, while great progress has been made in the bottom-up synthesis of GNRs with different structures^5, 6^, there were no experimental demonstrations of GNR-based devices with characteristics sufficiently good to justify a practical comparison of GNRs with benchmark electronic materials, including graphene. Already demonstrated GNR-based devices suffer from very-low currents and mobilities^7–12^. On one hand, there could be improvements on the device fabrication side, including smaller gaps between electrodes, lower contact resistances, thinner gate dielectrics, etc. On the other hand, there is a lot of room for improvement of the conductivity of GNR device channels. As discussed in theoretical studies^1, 13^, while the dependence of the bandgap of GNRs on their structural parameters is rather complex, the overall trend is that the bandgap decreases with increasing the ribbon width. Therefore, lateral extension of narrow GNRs could be a general approach to improve their electrical conductivity. Also, while some of the potential GNR-based devices could utilize individual ribbons bridging electrodes, others may use multiple GNRs that could be assembled in a form of thin films, fibers, single crystals, etc. For such devices based on nanoribbons assemblies, lateral extension of GNRs may also result in improved electrical conductivity due to better overlap between ribbons.

Several experimental studies utilized the idea of increasing the width of GNRs for decreasing their bandgaps^14, 15^. In this paper, we demonstrate how the lateral extension approach can be applied to chevron GNR, which is one of the most widely studied atomically precise GNRs. Chevron GNRs were first synthesized by the on-surface approach on Au(111) by Cai et al.^16^. Later, we demonstrated the solution synthesis of chevron GNRs by Yamamoto coupling of 6,11-dibromo-1,2,3,4-tetraphenyltriphenylene (C_42_Br_2_H_26_) followed by oxidative cyclodehydrogenation of the resulting polymer via Scholl reaction^17^. More recently, we developed a method for interfacial self-assembly of nanoribbons, with which we prepared very uniform thin films of chevron GNRs (cGNRs) that were used for the device fabrication^18^. Similarly to other device studies of atomically precise nanoribbons, field-effect transistors based on films of chevron GNRs exhibited low conductivities, mobilities, and on/off ratios.

Here we report the synthesis and characterization of laterally extended chevron GNRs (in this paper, we refer to them as eGNRs). We demonstrate that eGNRs can be processed into uniform thin films that have substantially higher electrical conductivity than similar films based on regular cGNRs. Thin films of eGNRs were effectively utilized in gas sensors that showed improved responsivity to low molecular weight alcohols compared to similar sensors based on benchmark graphitic materials, such as graphene and reduced graphene oxide (rGO). Finally, we employed eGNR films in an electronic nose system that could reliably recognize analytes of nearly the same chemical nature, such as methanol and ethanol.

## Results

### Design and computational study of eGNR

**Fig. 1:**
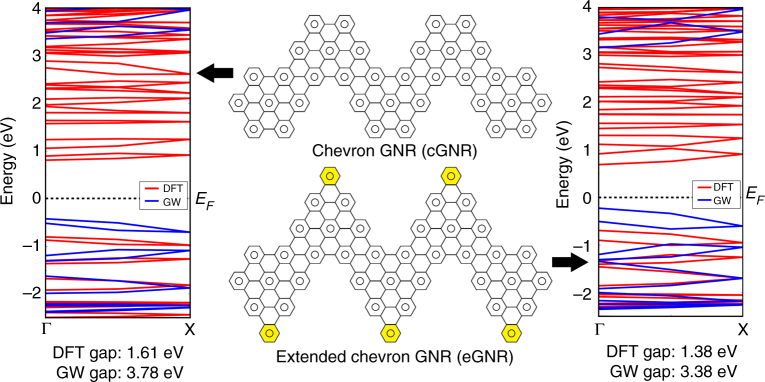
Comparison of regular chevron GNR (cGNR) and laterally extended chevron GNR (eGNR). The *middle panels* show fragments of atomic structures of cGNR and eGNR. eGNR can be viewed as cGNR with additional benzene rings (highlighted by *yellow*) to the elbow positions. The *side panels* show band structures for cGNR and eGNR, respectively, which were calculated using DFT (*red lines*) and GW (*blue lines*) methods

Figure 1 shows the molecular structure of the eGNR, which can be viewed as the regular chevron GNR with additional benzene rings (highlighted by yellow) in the elbow positions. Noteworthy, both cGNR and eGNR have Clar structures that are entirely described by aromatic π-sextet rings, suggesting high chemical stability of both these ribbons^19^.

Computational simulations reveal that the eGNR has a reduced bandgap compared with the original cGNR; see the corresponding band structures that were obtained using DFT and GW simulations in Fig. 1; computational details are provided in Supplementary Note 1. DFT results indicate that the 1.61 eV bandgap for the cGNR is reduced to 1.38 eV for the eGNR. More accurate GW simulations^20, 21^ were also performed and the results show that the value of the quasiparticle bandgap for eGNR is 3.38 eV, smaller than that of cGNR (3.78 eV). The DFT/GW calculated bandgap values (1.61/3.78 eV) that were obtained for the regular chevron GNR are in agreement with the previous study^22^.

### Synthesis and bulk spectroscopic characterization of eGNRs

**Fig. 2:**
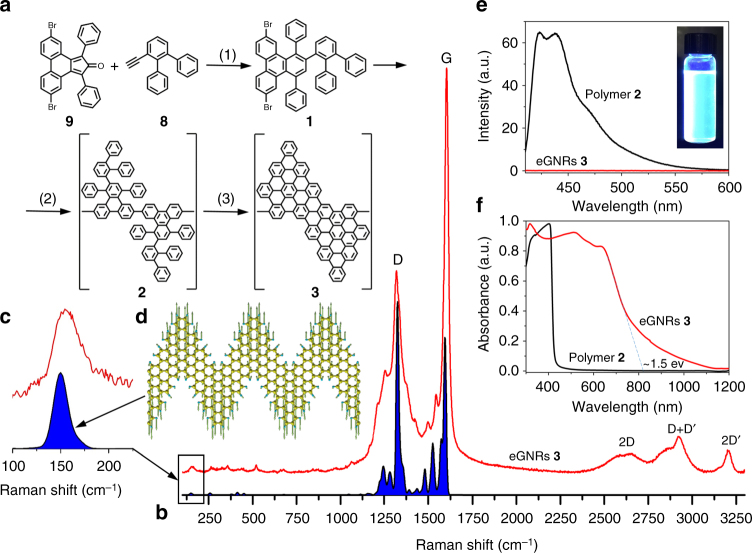
Synthesis and spectroscopic characterization of eGNRs. **a** Scheme of the solution synthesis of eGNRs: (1) Ph_2_O, reflux, 24 h; (2) Ni(COD)_2_, 1,5-Cyclooctadiene (COD), 2,2′-bipyridine, toluene, dimethylformamide, 75 °C, 72 h; (3) FeCl_3_, CH_3_NO_2_, dichloromethane, r.t., 48 h. **b** Experimental (*red*) and simulated using DFT (*blue*) Raman spectra of eGNRs. **c** Experimental (*red*) and simulated (*blue*) Raman spectra in the 100–200 cm^−1^ range showing the radial-breathing-like mode of eGNRs. **d** Scheme of the atomic displacements in eGNR that are characteristic for the radial-breathing-like mode at 149.75 cm^−1^. **e** Photoluminescence spectra of polymer **2** (*black*) and eGNRs **3** (*red*) recorded with a 405 nm excitation light. The inset shows an optical photograph of the photoluminescence of polymer **2** that was dispersed in dimethylformamide and illuminated by a 365 nm ultraviolet lamp. **f** UV-vis-NIR absorbance spectra of polymer **2** (*black*) and eGNRs **3** (*red*) suspended in dimethylformamide by sonication

The synthesis of eGNRs is schematically shown in Fig. 2a; the entire synthetic route is shown in Supplementary Fig. 1. In brief, we first synthesized compound **9** ^23^, which is a common precursor for chevron-type GNRs, including cGNRs^17, 24^ and nitrogen-doped chevron GNRs^25, 26^. Then, we synthesized precursor molecule **1** through the Diels-Alder reaction between compound **9** and 3′-ethynyl-1,1′:2′,1″-terphenyl **8**; the complete procedure for the synthesis of molecule **1** is described in detail in Supplementary Note 2. Then, molecule **1** was polymerized using Ni^0^-mediated Yamamoto coupling, forming polymer **2**. Finally, eGNRs **3** were produced by oxidative cyclodehydrogenation of polymer **2** via the Scholl reaction using iron(III) chloride. Nuclear magnetic resonance (NMR) data for compounds **1**, **2** and **3** are shown in Supplementary Figs 2–4. The resulting eGNRs were washed from impurities, isolated as a black powder and then used for materials characterization and device fabrication.

Polystyrene (PS) calibrated size-exclusion chromatography (SEC), is a commonly used characterization technique for GNR precursor polymers^14, 27–30^, and was employed to characterize polymer **2**, see Supplementary Fig. 5. The weight average molecular weight, *M*_w_ = 3.0 × 10^4^ g mol^−1^ corresponded to ~ 50 debrominated monomer units. This compares to an average polymer length of 42.5 nm. The polydispersity index (PDI) of these eGNR precursor polymers is 3.2. The actual molecular weight is usually overestimated when polystyrene is compared to more rigid polymers^31, 32^.

Raman spectroscopy is a very powerful tool for studying carbon nanomaterials^33, 34^. Figure 2b demonstrates a Raman spectrum of eGNRs. In addition to the most intense lines at 1318 and 1603 cm^−1^ that are commonly observed for *sp* ^2^ carbon materials and typically referred to as D and G bands, respectively^34^, numerous smaller peaks can be seen (Fig. 2b). We performed the DFT simulations of the Raman spectrum of eGNR (the details of simulations are given in Supplementary Note 3) and found a good agreement between the calculated and experimentally observed peaks. In accordance with the experiment, the calculated spectrum predicts several peaks at the left shoulder of the D band, and in between the D and G bands, but no additional peaks at the right shoulder of the G band. Several small Raman peaks are also observed at low frequencies. Similarly to single-walled carbon nanotubes, which exhibit radial breathing modes (RBMs) that are inversely proportional to the tube’s diameter^35^, atomically precise GNRs were also shown to have analogous low-frequency modes that are often referred to as RBM-like modes^16, 30, 36^. We analyzed the atomic displacements for different low-frequency Raman peaks predicted for eGNR by the DFT simulations, and found that the RBM-like mode for this ribbon should appear at 149.45 cm^−1^ (Fig. 2c); the actual displacement pattern for this vibration is shown in Fig. 2d. Remarkably, the experimental Raman spectrum shows a distinct low-frequency peak in a very good agreement with theoretical predictions (Fig. 2c). Overall, given the high sensitivity of Raman spectroscopy to disorder in carbon materials^33, 34^, the high quality of Raman spectra of eGNRs and the close agreement between experimental and theoretical data confirm the high structural quality of eGNRs.

Figure 2e, f demonstrates photoluminescence and UV-vis-NIR data for eGNRs as well as the polymer **2**; these results are similar to those previously reported for chevron GNRs and their precursor polymer^17^. Figure 2e demonstrates that while polymer **2** shows a bright blue emission, eGNRs exhibit no visible photoluminescence. Figure 2f shows UV-vis-NIR spectra of eGNRs and polymer **2** dispersed in dimethylformamide. Cyclization of yellow polymer **2** results in the formation of the extended aromatic system that absorbs visible range photons, and the color changes from yellow to black. The eGNR spectrum exhibits a strong absorption in the UV and visible region and an absorption edge in NIR. The extrapolation to zero of the linear part of the absorption decay gives an optical bandgap of eGNRs of ~ 1.5 eV, while for solution-synthesized cGNRs the results of UV-vis-NIR and photoluminescence spectroscopy suggest an optical bandgap of 1.6–1.8 eV^24, 26^. Overall, the slight reduction of the optical bandgap is consistent with the calculated band structures of eGNRs and cGNRs (Fig. 1).

### Scanning tunneling microscopy and spectroscopy of eGNRs

Ultrahigh vacuum (UHV) scanning tunneling microscopy (STM) was used to confirm the structure of the eGNRs and measure their bandgap. In our original study on the solution synthesis of cGNRs, we visualized nanoribbons by drying a droplet of a cGNR suspension on the Au(111) surface, transferring the substrate to a UHV chamber and performing STM imaging^17^. While high-resolution STM images of cGNRs were demonstrated, the downside of this approach was that even in UHV conditions it is difficult to entirely remove the residual solvent molecules and other contaminants that adsorb on the Au (111) surface during the sample preparation in air. Recently, we demonstrated an alternative sample preparation approach, the dry contact transfer (DCT) method, by which it is possible to prepare cleaner samples of solution-synthesized GNRs for STM analysis^37^. In brief, in this method a GNR powder is annealed in UHV to remove adsorbates and solvent residues, and then pressed against an already prepared, clean substrate for STM imaging using a fiber-glass applicator. As a result, some GNRs from the powder are transferred to the substrate directly in the UHV environment, and no solvents are involved in this process.

**Fig. 3:**
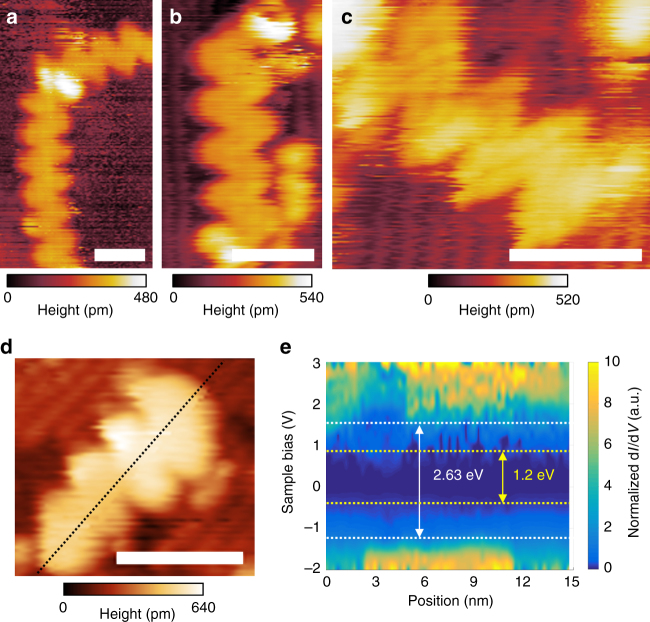
STM and STS characterization of eGNRs. **a**–**c** STM images of eGNRs on InAs(110). *Scale bars* are 3 nm. Scan parameters: −2 V, 8 pA. **d** STM image of eGNR on H:Si(100). *Scale bar* is 4 nm. Scan parameters: −2.5 V, 10 pA. **e** Normalized d*I*/d*V* map along the dashed line in **d** showing a  ~ 2.63 eV bandgap over the eGNR and a ~ 1.2 eV bandgap over the silicon substrate, which is in close agreement with the expected 1.1 eV value

In the present study, we used the DCT method to transfer eGNRs onto two semiconductor substrates, InAs(110) and H:Si(100). A detailed description of the STM instrumentation, as well as the DCT process, can be found in our previous work^37^, in which we studied cGNRs transferred onto H:Si(100). Here we also employed the InAs(110) surface, which was chosen for its high surface energy to help immobilize the eGNRs during imaging and characterization at room temperature. On both substrates we have obtained STM images of eGNRs, which confirm their high structural quality (Fig. 3). Figure 3a–c shows STM images of isolated eGNRs deposited onto cleaved InAs(110); the ribbons look flat and well cyclized. The underlying As sublattice is clearly visible, and in Fig. 3c some features corresponding to the graphene nanoribbon electronic structure are resolved, which indicates a clean transfer.

It should be noted that in a powder form eGNRs are heavily aggregated, which can be explained by their entangling and π-π stacking. Supplementary Fig. 6 shows that the eGNR powder consists of dense particles with sizes up several tens of μm. When they are pressed against a semiconductor substrate, it is more likely that shorter eGNRs that are less entangled and weaker bound to other nanoribbons will be exfoliated from the aggregates to a surface easier than the longer ones. This can explain why the mean length of 14.8 nm, which we measured for 20 eGNRs observed on both InAs(110) and H:Si(100) substrates, was smaller than the average polymer length of 42.5 nm obtained from the SEC measurements. It should also be noted that when eGNRs are torn from a particle and exfoliated onto a substrate, they should not necessarily be perfectly flat on a surface, but may rather contain twists and wrinkles. For example, the white spot on the eGNR in Fig. 3a, which corresponds to a larger topographic height, may be associated with a structural defect, but may as well be a wrinkle that is responsible for the change in the ribbon’s direction.

We also compared STM images of cGNRs and eGNRs, Supplementary Fig. 7. Both GNRs show the characteristic chevron geometry, and the height profiles measured along the long axes of nanoribbons show that cGNRs and eGNRs have the same structural period of ~1.7 nm (Supplementary Fig. 7c, d), which is expected from their atomic structures (Fig. 1). The average apparent heights of these two GNRs were also comparable, 0.25 nm for eGNRs and 0.28 nm for cGNRs, Supplementary Fig. 7e, f (note that the measurements were performed on different semiconductor substrates, InAs(110) and H:Si(100), respectively). The height profiles also indicate the apparent width across the eGNR of 2.4 nm, which is larger than the 2.2 nm apparent width measured for the cGNR. Although these values are affected by the tip convolution effect, the increase in the apparent width is consistent with the laterally extended structure of the eGNR. In addition, STM measures electronic features and not the atomic positions so the extension of the local density of states increases the observed width.

For eGNRs deposited on H:Si(100) we performed scanning tunneling spectroscopy (STS), whereby we collected *I–V* spectra and numerically calculated the normalized tunneling conductance to examine the local density of states of the ribbons. STS of eGNRs was performed on H:Si(100) to allow direct comparison to the recent study of cGNRs on H:Si(100)^37^. Figure 3d shows STM image of a short eGNR on H:Si(100) substrate, for which STS measurements were performed along the dashed line; the results are demonstrated in Fig. 3e showing the bandgap of ~ 2.63 ± 0.2 eV (see Supplementary Fig. 8 for information regarding the bandgap determination). Together with STS results obtained on other eGNRs on H:Si(100) (one additional set of STM/STS data for a different eGNR on H:Si(100) is shown in Supplementary Fig. 8) we found an average eGNR bandgap of 2.66 ± 0.5 eV. In comparison, a similar analysis of STS measurements performed for solution-synthesized chevron GNRs on H:Si(100) reveals a bandgap of ~ 2.76 ± 0.3 eV.

Both optical and STS bandgaps of eGNRs are smaller than the theoretically predicted bandgap determined with the GW approximation (3.38 eV, Fig. 1), which is consistent with similar data obtained for other atomically precise GNRs^38, 39^. The bandgap measured with STS is smaller than the GW gap due to a surface polarization effect^38–40^, while the observed optical bandgap is smaller than the theoretical gap because of the excitonic contribution^38^. Yet, both optical and STS measurements reveal that eGNRs have a slightly smaller bandgap than cGNRs, which is consistent with the results of computational simulations (Fig. 1) and the overall anticipated effect of lateral extension.

### Electrical and sensor measurements of eGNRs

**Fig. 4:**
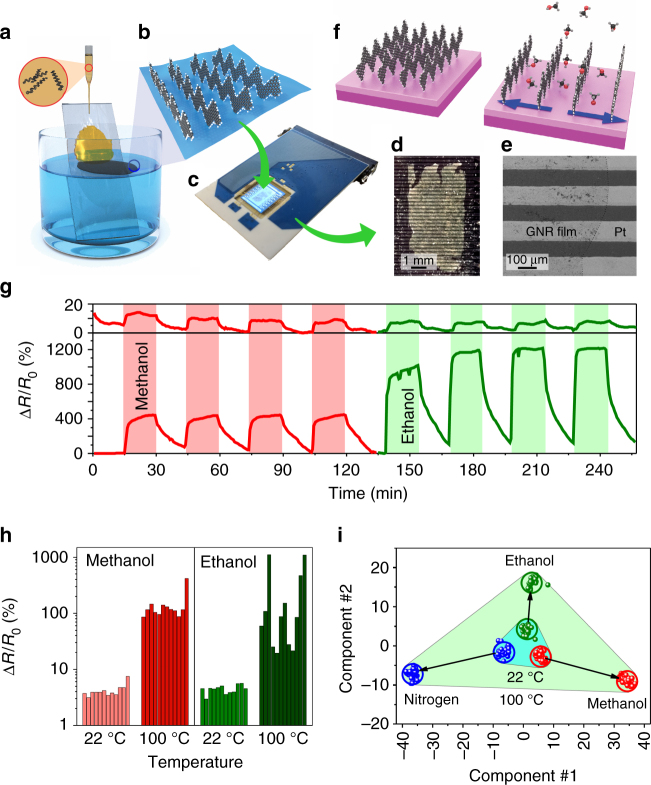
eGNR-based gas sensors. **a** Scheme of the interfacial self-assembly of GNRs; see text for details. **b** Scheme of the self-assembly of GNRs on the water surface. **c** Optical photograph of a multielectrode KAMINA chip with a self-assembled eGNR film. **d** Optical photograph of the active area of the KAMINA chip. The bright horizontal lines are Pt electrodes and the rectangle in the center is the eGNR film. **e** SEM image of the fragment of the eGNR film on the multielectrode structure. The edge of the eGNR film is shown by the dotted curve. **f** Scheme of the possible intercalation of alcohol molecules that may contribute to the decrease in the conductivity of the eGNR film; see text for details. **g** Representative dynamic responses of a selected segment of an eGNR sensor array to 500 p.p.m. methanol and ethanol at room temperature (22 °C) and at 100 °C. Colored vertical stripes indicate periods of time when the sensor was exposed to analytes. **h** Sensor responses (Δ*R*/﻿*R*_0_, %) of 12 representative eGNR sensor segments to 500 p.p.m. ethanol and methanol, respectively, at room temperature (22 °C) and 100 °C. **i** The results of LDA processing of the responses generated by the array of eGNR sensors that were exposed to nitrogen mixed with 500 p.p.m. of ethanol and methanol at 22 °C (*small blue triangle*) and 100 °C (*large green triangle*)

The results of bulk measurements of cGNRs and eGNRs also suggest that lateral extension of nanoribbons results in their stronger overlap. As we discuss in Supplementary Note 4 and show in Supplementary Fig. 9, pressed pellets of eGNR powder have higher density compared to similarly prepared pellets of cGNRs, and particles of eGNRs are more electrically conductive compared to similar particles of cGNRs. However, more device relevant is the comparison of electrical conductivities of thin films of eGNRs and cGNRs. In order to fabricate thin films of cGNRs and eGNRs we used the recently developed interfacial self-assembly approach^18^ (Fig. 4a). This method takes advantage of the high solubility of non-functionalized GNRs in chlorosulfonic acid (CSA), while they are insoluble in most other solvents. When a droplet of a GNR solution in CSA is delivered to the surface of water, CSA reacts instantly with H_2_O forming sulfuric and hydrochloric acids that are dissolved in water, leaving hydrophobic GNRs at the polar water-air interface. The GNRs, which are insoluble in water, minimize their interaction with the water molecules by forming a densely packed self-assembled film of π–π stacked nanoribbons, as shown in Fig. 4b. The GNR film can be washed from the CSA residues by transferring it to the surface of deionized (DI) water (this procedure is repeated several times) and eventually fished out with a substrate of choice^18^. In this study, the self-assembled films of cGNRs and eGNRs were fished out from the surface of DI water with multielectrode KAMINA chips, one of which is shown in Fig. 4c ^41^. Previously, we used KAMINA chips to measure electronic and sensor properties of other graphitic materials, such as rGO^42^ and graphene^43^. Figure 4d shows the active area of the chip, which consists of an 8 × 10 mm^2^ Si/SiO_2_ substrate with 39 Pt electrodes (100 × 3000 μm^2^ each) that are separated by  ~ 70 μm gaps, and two Pt thermoresistors. When deposited on a KAMINA chip, the GNR film (a bright rectangle in the center of Fig. 4d) bridges the Pt electrodes, thus forming 38 two-terminal GNR devices that can be measured independently. Figure 4e shows scanning electron microscopy (SEM) image, in which the edge of the eGNR film bridging several Pt electrodes can be seen. The back side of the Si/SiO_2_ substrate contains four independent Pt meander heaters, which allow electrical measurements at elevated temperatures.

We compared electrical conductivities of cGNR and eGNR films that were prepared by the same approach^18^ (Fig. 4a–c) and transferred to KAMINA chips. The eGNR films were more than an order of magnitude more electrically conductive than films of cGNRs with a sheet resistance of ~ 1×10^9^ Ω/sq versus ~ 2×10^10^ Ω/sq; this difference is in agreement with the results of electrical conductivity measurements of individual particles of eGNRs and cGNRs that is discussed in Supplementary Note 4. While the conductivity of eGNR films was still rather low, it was sufficient for performing the gas sensor measurements using the setup described in Supplementary Note 5.

Previously, we used KAMINA chips to measure electronic and sensor properties of other graphene materials, such as rGO^42^ and graphene films grown by chemical vapor deposition (CVD)^43^. In those studies, we tested the sensor responses of rGO and CVD graphene to various alcohols, such as methanol, ethanol and isopropanol^42, 43^. Therefore, for the sake of comparison of atomically precise GNRs with important graphene materials that are commonly used in sensor studies^44^, we performed similar sensing experiments with eGNRs. The details of sensor measurements are provided in Supplementary Note 5 and the experimental setup is shown in Supplementary Fig. 10. Supplementary Fig. 11 shows that (a) eGNR devices exhibit linear *I–V* curves, suggesting Ohmic contacts between eGNR films and Pt electrodes, and (b) there is an almost linear dependence of the responses of eGNR segments on analyte concentrations, suggesting that the channel material rather than contact resistance is responsible for the observed sensor responses.

In general, when analytes adsorb on graphene, its electrical conductivity changes because of charge doping by the molecules^45^. More specifically, when rGO or CVD graphene sensors are exposed to low molecular weight alcohols, their resistance increases^42, 43^. The same was observed for eGNRs sensors, but the magnitude of the effect was much greater. Segments of KAMINA chips covered with rGO films or CVD graphene changed their resistance upon exposure up to 1500 p.p.m. of ethanol or methanol by only a few percent^42, 43^. Recently reviewed gas sensors based on various nanocarbon materials show comparable responses to a large variety of analytes^46^. In contrast, in this work on eGNRs sensors we observed resistance changes of several hundred percent; for instance, the resistance of some of the eGNR segments grew by over 1200% when they were exposed to 500 p.p.m. ethanol at 100 °C. This could be rationalized as follows. In addition to the same charge doping effect as in the case of other graphitic materials, there could be another mechanism of resistance increase upon exposure of eGNR sensors to analyte molecules.

Since the gaps between Pt electrodes on a KAMINA chip are about 70 μm, while individual eGNRs are significantly shorter, eGNR film devices in this experiment contained multiple inter-ribbon junctions, and their contributions to the electron transport should be very important. As we discussed previously^18^, hydrophobic GNRs self-assemble in the edge-on geometry on the surface of water (Fig. 4b) and this arrangement, which is stabilized by π-π interactions between nanoribbons, remains after transfer of a GNR film to a solid substrate. As a result, the interplanar spacings between eGNRs are accessible to the analyte molecules that may intercalate between nanoribbons. As shown in Fig. 4f, such intercalation should increase the interplanar spacings between eGNRs, which would impede the charge transfer between nanoribbons and decrease the overall electrical conductivity of the eGNR film.

The experimental observations agree with the proposed mechanism (Fig. 4f). First of all, the sensor response of eGNR films to alcohols greatly increases at elevated temperatures, as illustrated by Fig. 4g, h. Figure 4g shows representative data for dynamic sensor responses of one of the eGNR devices in a multisensor array that was measured at room temperature and 100 °C, while Fig. 4h shows relative resistance changes (Δ*R*/*R*_0_) of 12 different eGNR segments. Note that the same segments are shown in the same order for methanol and ethanol at both temperatures, and the segments were chosen to show both minimum and maximum responses. These results can be rationalized by the fact that at high temperature, the increased interplanar spacings between eGNRs and higher kinetic energies of analyte molecules should both facilitate the intercalation.

The analysis of the responses of different sensors, especially at high temperature, further suggests the importance of the morphological features of eGNR segments for their sensor behavior. Figure 4h shows that at room temperature the responses of 12 eGNR segments to both methanol and ethanol are comparable. However, the situation changes at 100 °C, where the signals remain fairly uniform in case of the methanol sensing but vary considerably for the ethanol exposure. Remarkably, some sensors show higher responses to methanol than to ethanol, while others show the opposite behavior. This cannot be explained solely by the doping of eGNRs by the methanol and ethanol molecules, which would cause the same trend for all devices when switching from methanol to ethanol. However, as we discussed in our previous study^18^, the morphology of the eGNR channels of different segments could vary considerably, because the film consists of randomly oriented domains of π-π stacked nanoribbons and nanoscopic holes. These morphological differences between different segments are likely to contribute to the local kinetics of adsorption and intercalation of alcohol molecules and thus to the observed device-to-device variability. Noteworthy, no difference in the range of Δ*R*/*R*_0_ values was observed for these analytes in experiments with similar rGO and CVD graphene sensors^42, 43^—for those sensors, the intercalation effects should not be as important as for the devices made of vertically stacked eGNRs, while the doping effects of methanol and ethanol were comparable.

Since both methanol and ethanol cause resistance increase in eGNR devices, differentiation between these analytes using a single eGNR sensor could be a difficult task. However, the selectivity toward similar analytes can be achieved by employing an array of sensors that have a large device-to-device variability. A large array of such sensors, for which the data are processed using pattern recognition algorithms could be considered as an electronic nose or e-nose; see review papers^47, 48^ and references therein. E-nose systems demonstrate very high selectivity in analyte recognition: although the intrinsic selectivity of a sensing material may be low, the combination of several segments in an array has a very large information content. An e-nose system is first calibrated to create a library of analytes of interest, and in the following recognition experiments, the measured analyte signals are compared with ones recorded in the library^49^. For the best performance of an e-nose system, the segments of an array should exhibit a substantial variability in their sensor properties.

As we show in Fig. 4h, the eGNR segments of a KAMINA chip naturally have a large device-to-device variation because they consist of randomly oriented domains of π-π stacked ribbons with different lengths. Additionally, the transfer process may produce macroscopic cracks, tears and holes in eGNR film and thus contribute to the variability between eGNR device channels in different segments. In our study, the sensor responses of different eGNR segments to the same analyte at the same conditions could vary by up to two orders of magnitude (see, Δ*R*/*R*_0_ values in the experiment on ethanol sensing at 100 °C). Therefore, the multisensor KAMINA chip covered with an eGNR film can be directly employed as an e-nose system.

The observed variability in sensor responses of eGNR segments was sufficient to reliably discriminate methanol and ethanol at concentrations of 500 p.p.m., as well as nitrogen. We processed the sensor responses, excluding the first exposure-purge cycles, by the pattern recognition technique based on Linear Discriminant Analysis (LDA)^50^. This technique transfers the multidimensional sensor signals to a reduced two-dimensional space where the sensor responses of eGNR segments are grouped into separate clusters representing different analytes. The components 1 and 2 in Fig. 4i are mathematically constructed from the sensor responses using the LDA approach to maximize the distances between vectors corresponding to different clusters and thus ensure the reliable gas recognition^50^. Figure 4i shows the results of the LDA processing of the data from the eGNR-based multielectrode chip with the confidence probability of 0.95 at room temperature and at 100 °C. The results demonstrate the capability of the eGNR sensor array to reliably discriminate analytes of nearly the same chemical nature, such as the low molecular weight alcohols used in this study. Because of the higher sensor responses and their larger variability at higher temperatures, the discrimination of methanol and ethanol is considerably more efficient at 100 °C (large green triangle in Fig. 4i) than at room temperature (small blue triangle in Fig. 4i).

## Discussion

In summary, in this study we explored the idea of lateral extension of atomically precise GNRs for improving their electrical conductivity. We started with a widely studied chevron GNR, and designed a new laterally extended chevron GNR or eGNR (Fig. 1). The synthesis of eGNRs was supported by a number of techniques, including high-resolution STM and Raman spectroscopy supported by computational analysis. Optical spectroscopy and STS confirmed that lateral extension of chevron GNRs resulted in a decrease in their electronic bandgap. As expected, the macroscopic assemblies of eGNRs showed improved electrical conductivity compared to regular chevron GNRs, which is likely due to not only their smaller bandgap, but also the improved overlap between nanoribbons due to their extended width. The electrically conductive films of eGNRs were employed in gas sensors that showed improved responsivity to low molecular weight alcohols, compared to similar sensors based on widely studied graphene materials, such as rGO and CVD graphene^42, 43^. Finally, we demonstrated the first e-nose system based on atomically precise GNRs that exhibited reliable recognition of analytes of nearly the same chemical nature, methanol and ethanol.

This study opens multiple directions in research on atomically precise GNRs. Other designs of laterally extended chevron GNRs and other kinds of previously studied atomically precise GNRs can be considered, some of which may show further improvement in electrical conductivity and/or sensor properties. The study of the sensor properties of eGNRs is preliminary in a sense that we did not test any analytes other than low molecular weight alcohols—larger sensor responses and better recognition may be found for other molecules. Finally, this study suggests that other electrically conductive graphitic building blocks, such as certain polycyclic aromatic hydrocarbon (PAH) molecules, assembled in the edge-on geometry, may hold promise for sensor applications.

## Methods

### Synthesis of 2-([1,1’:2’,1”-terphenyl]-3’-yl)-6,11-dibromo-1,4-diphenyltriphenylene (1)

Synthesis of the eGNR precursor (molecule **1**) is described in detail in Supplementary Note 2. The scheme of the synthesis is shown in Supplementary Fig. 1.

### Synthesis of polymer (2)

Bis(1,5-cyclooctadiene)nickel(0) (0.5 g, 1.82 mmol), 2,2′-bipyridyl (0.284 g, 1.82 mmol), and 1,5-cyclooctadiene (223 µl, 1.82 mmol) were added to anhydrous dimethylformamide (6 ml). The reaction mixture was heated to 60 °C and kept for 30 min. Then 2-([1,1′:2′,1"-terphenyl]-3′-yl)-6,11-dibromo-1,4-diphenyltriphenylene **1** (0.8 g, 1.04 mmol) dissolved in anhydrous toluene (15 ml) was added to the reaction flask. The reaction was heated to 76 °C and kept for three days. After the reaction was allowed to cool to room temperature, methanol was added to precipitate the polymer. It was filtered, washed with methanol, concentrated hydrochloric acid, water, potassium hydroxide (1 M) in methanol, water, acetone, and hexane to obtain the title compound as a yellow solid (0.455 g, 71.9% yield).

### Synthesis of eGNR (3)

Dichloromethane (90 ml) was degassed with nitrogen bubbling for 15 min. Polymer **2** (40 mg, 65.7 µmol) and iron(III) chloride (0.45 g, 2.77 mmol) dissolved in nitromethane (5 ml) were added to the reaction mixture. The reaction was stirred for two days with nitrogen being bubbled through the reaction continuously. The reaction mixture was filtered, washed with concentrated hydrochloric acid, potassium hydroxide (1 M) in methanol, methanol, and acetone to obtain the title compound as a black solid (32 mg, 81.6% yield).^13^C NMR spectra of the polymer **2** (red) and eGNRs **3** are shown in Supplementary Fig. 4.

### Sample characterization

^1^H and ^13^C NMR was performed on Bruker 400, 600, and 700 MHz instruments. Magic angle spinning was performed at 600 MHz with the spinning speed of 8 kHz.

Raman spectrum of GNRs was recorded using a Thermo Scientific DXR Raman Microscope with a 532 nm laser.

UV-vis-NIR spectroscopy was performed on a Jasco V-670 Spectrophotometer. Photoluminescence (PL) spectra were obtained using a Shimadzu RF-5301PC instrument using a 400 nm excitation light. For UV-vis-NIR and PL measurements of polymer **2** and eGNRs **3**, approximately 0.5 mg of the corresponding material was added to 5 ml of dimethylformamide. The suspension was sonicated for 30 min before the measurements.

Size-exclusion chromatography was performed with an Agilent 1260 Infinity instrument. Approximately 10 mg of polymer **2** was dissolved in 15 ml of tetrahydrofuran. The sample was sonicated for 1 h and allowed to swell overnight. Column and detector temperature was set to room temperature. Injection volume was 10 µl. Polystyrene standards with narrow molecular weight distributions were used to calibrate the system. A PLgel 5 µm MIXED-D column with a PLgel 5 µm guard were used.

STM and STS characterization of nanoribbons was performed using a home-built STM instrument. A detailed description of the instrument, sample preparation and imaging conditions can be found in our recent work^37^.

Scanning electron microscopy images were taken using a Zeiss Supra 40 field-emission scanning electron microscope.

The details of electrical measurements are given in Supplementary Note 4. The setup for gas sensing measurements is shown in Supplementary Fig. 10 and described in Supplementary Note 5; we used the same setup for the sensor studies of other graphene materials, such as rGO and CVD graphene^42, 43^.

### Data availability

The data that support the findings of this study are available from the corresponding author upon request.

## Electronic supplementary material

Supplementary Information
