# Supplementary material for: Laterally extended atomically precise graphene nanoribbons with improved electrical conductivity for efficient gas sensing
Source: Nat Commun. 2017 Oct 10;8:820. doi: 10.1038/s41467-017-00692-4 (PMC5635063; doi:10.1038/s41467-017-00692-4)
Supplement: Supplementary file 1 — Supplementary Information [file 41467_2017_692_MOESM1_ESM.pdf]

Description of Supplementary Files

File Name: Supplementary Information
Description: Supplementary Figures, Supplementary Notes and Supplementary References

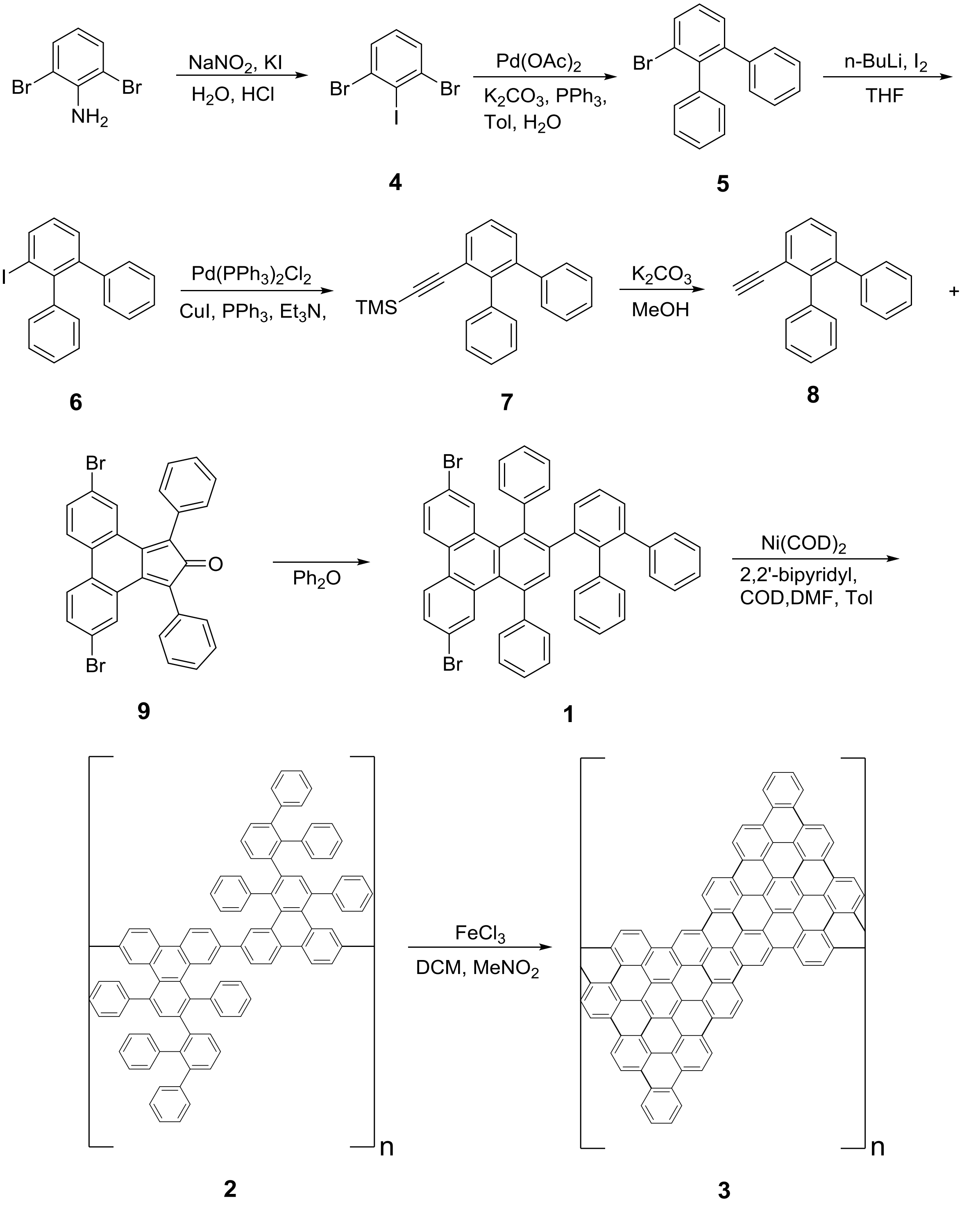

Supplementary Figure 1. Synthetic route to eGNR **(3)**. Procedures for the synthesis of molecule **(1)** are described in Supplementary Note 2. Procedures for the solution polymerization of molecule **(1)** to form polymer **(2)**, and the conversion of polymer **(2)** to eGNR **(3)** are given in the Methods section in the main text.

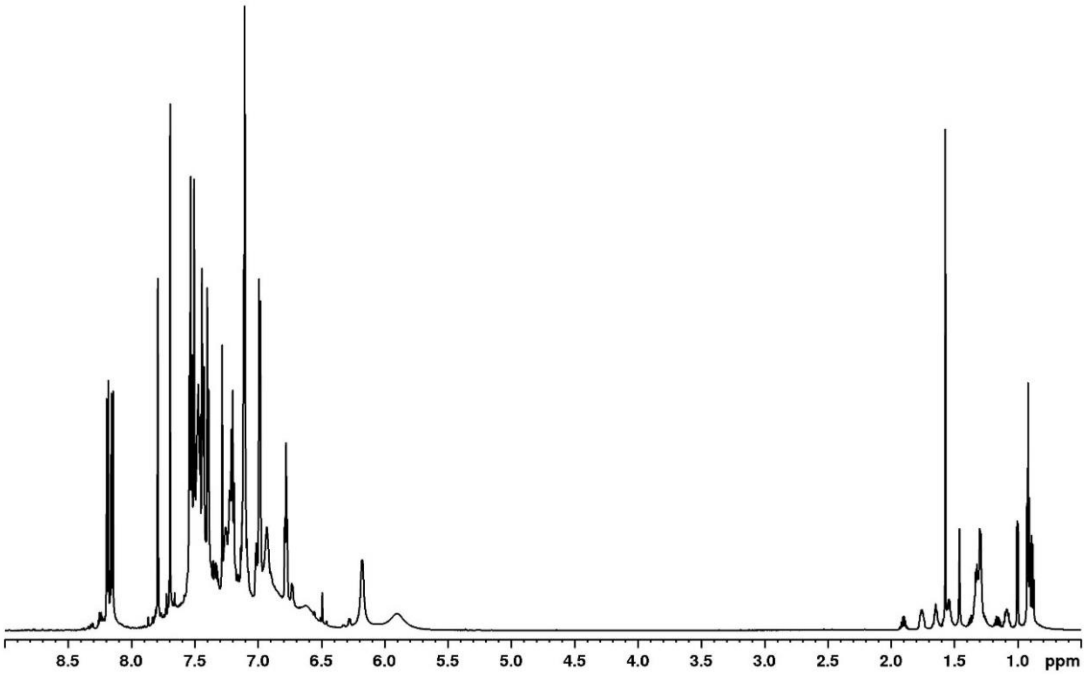

Supplementary Figure 2. ^{1}H NMR spectrum of 2-([1,1':2',1''-terphenyl]-3'-yl)-6,11-dibromo-1,4-diphenyltriphenylene **(1)** in $CDCl_3$.

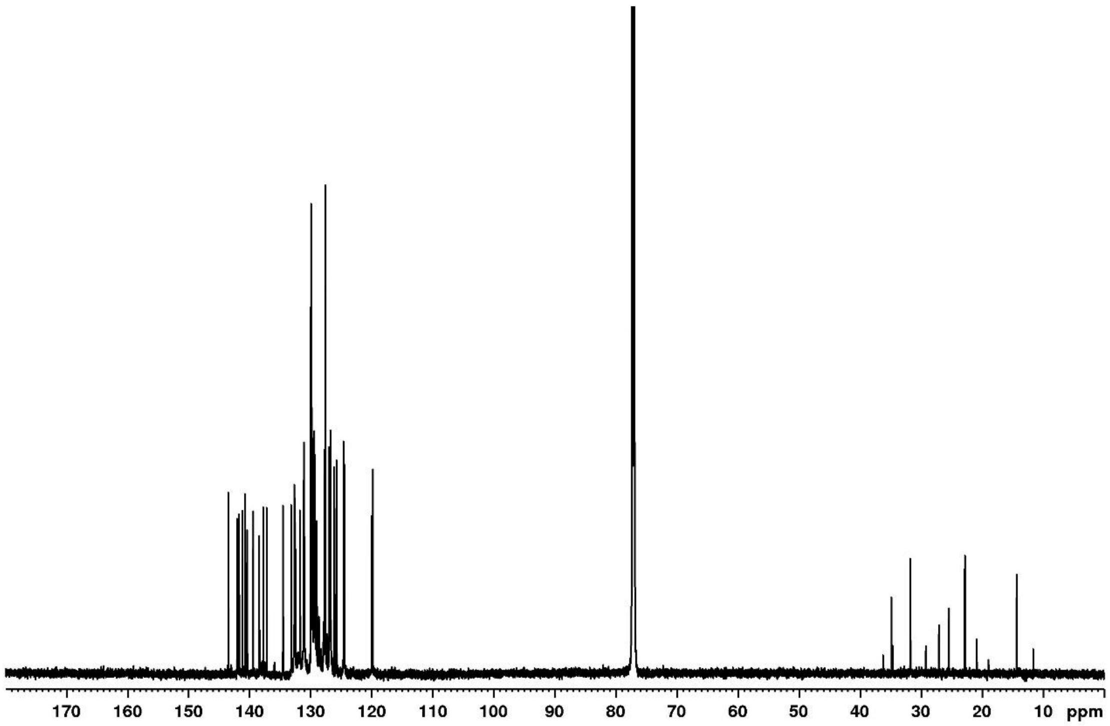

Supplementary Figure 3. ^{13}C NMR spectrum of 2-([1,1':2',1''-terphenyl]-3'-yl)-6,11-dibromo-1,4-diphenyltriphenylene **(1)** in $CDCl_3$.

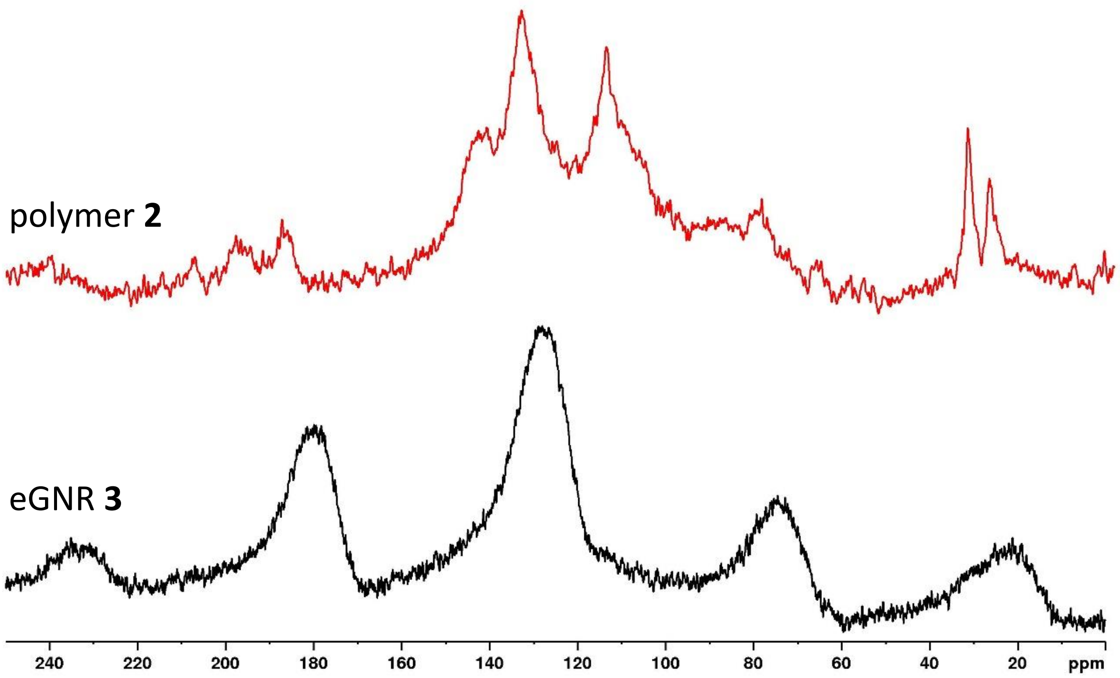

Supplementary Figure 4. ^{13}C solid state NMR spectra of polymer **(2)** (red) and eGNR **(3)** (black).

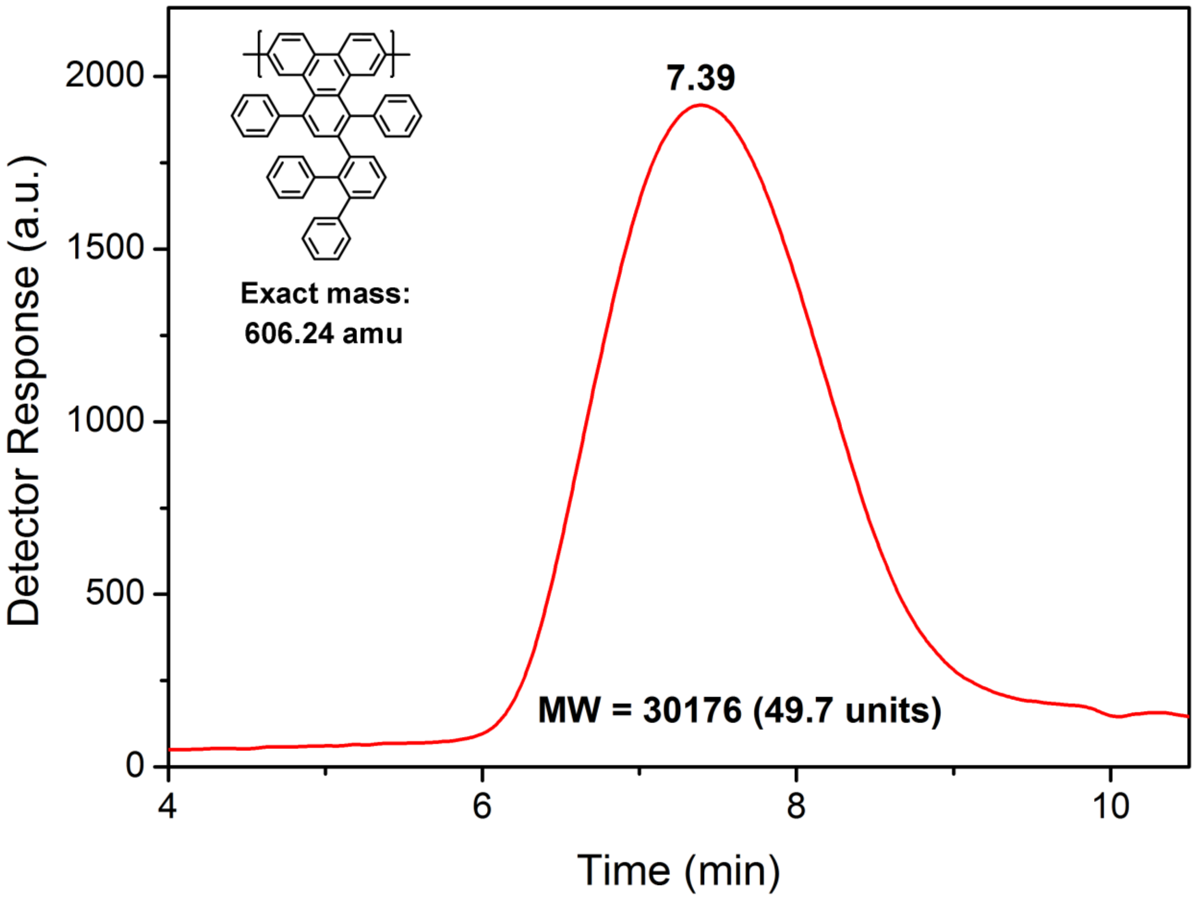

Supplementary Figure 5. Size-exclusion chromatography pattern of polymer **(2)** in THF. Normalization against polystyrene standards gives a weight average molecular weight (M_w) of 3.0×10^4 g mol^{-1}. Eluent: THF, 1.0 mg/mL, RI detector.

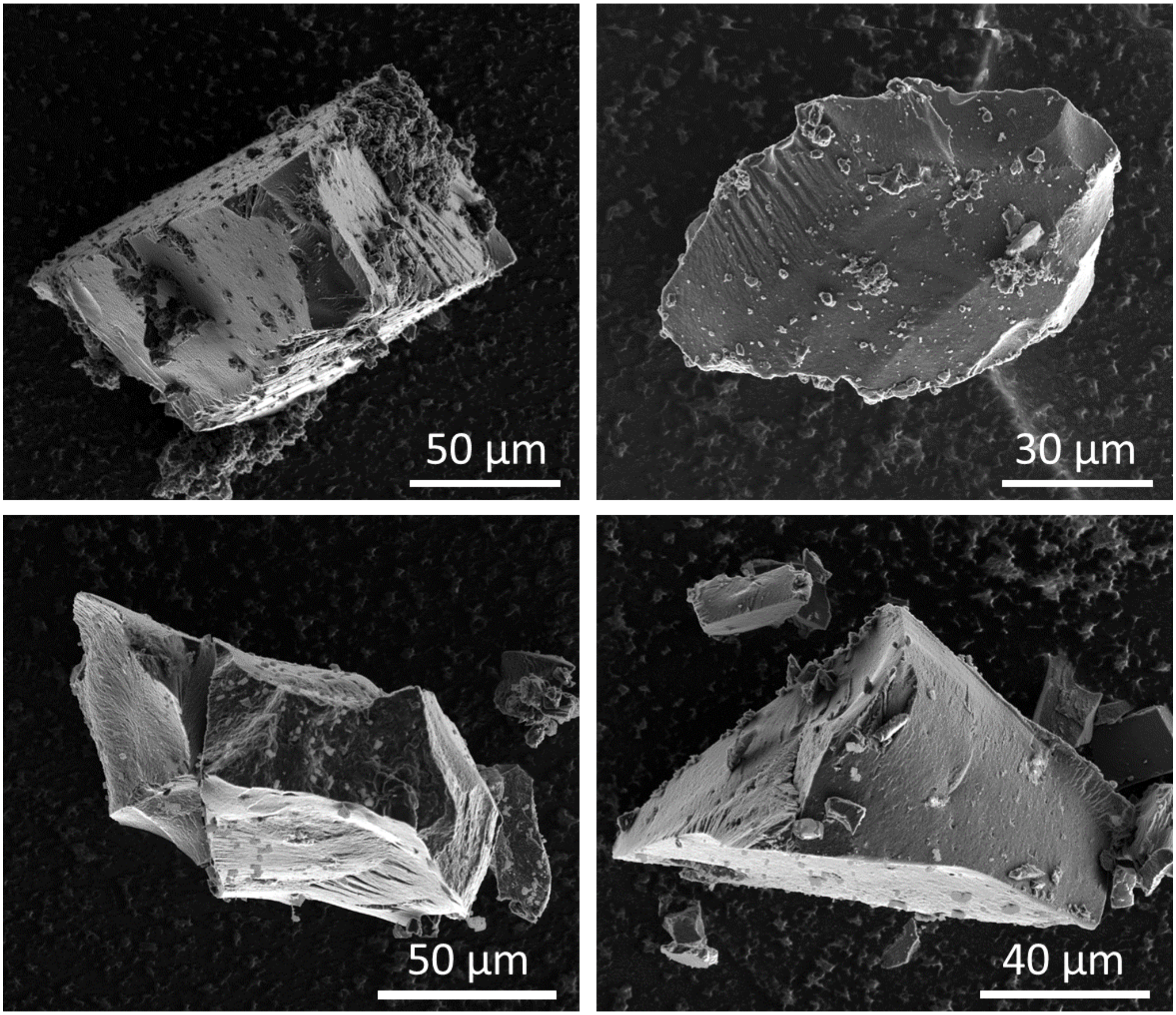

Supplementary Figure 6. SEM images of eGNR aggregates on a conductive carbon tape. A powder consisting of such particles was used in the DCT process to prepare samples for STM/STS characterization of eGNRs.

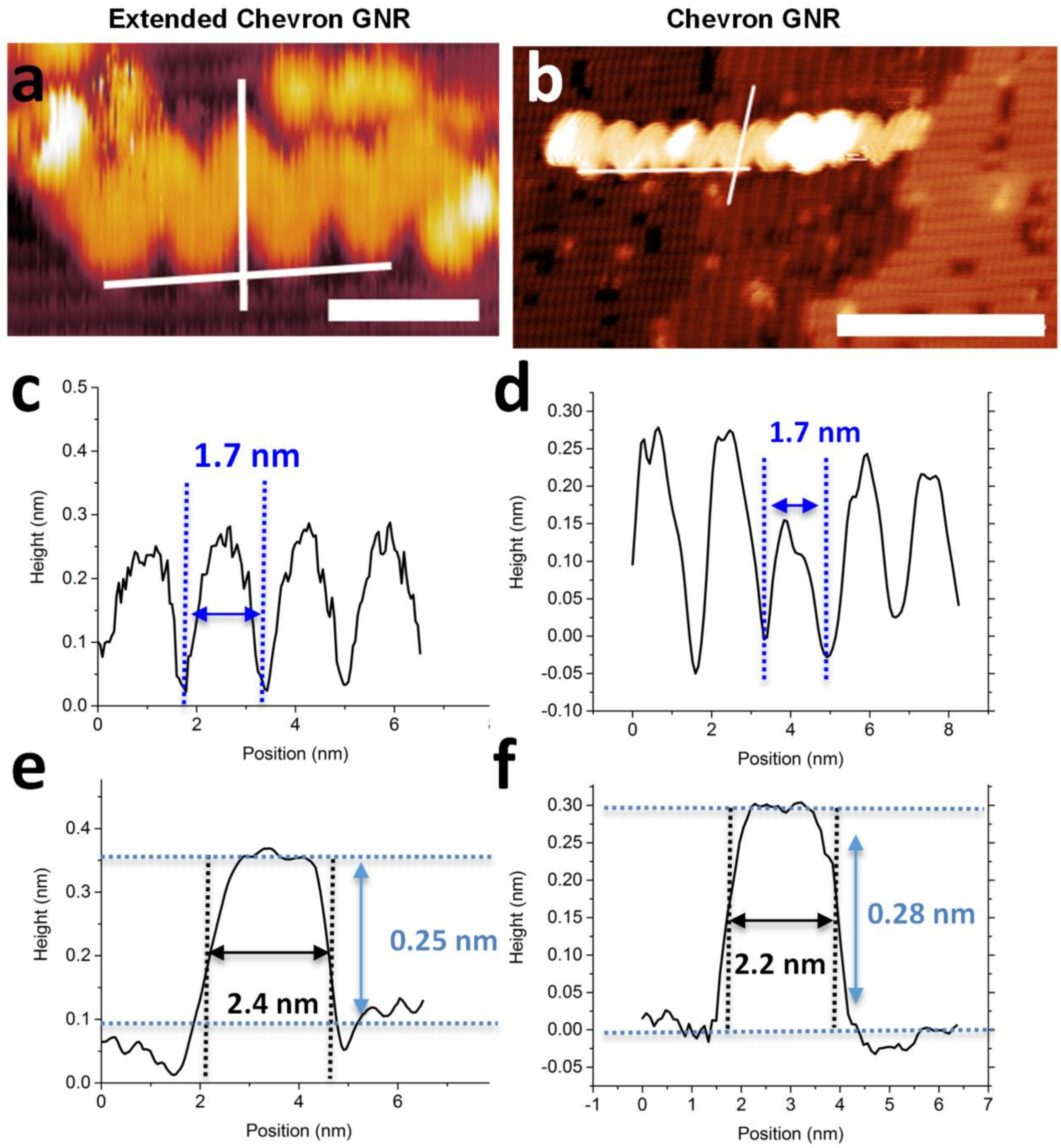

Supplementary Figure 7. Comparison of STM images of eGNR and cGNR. **(a)** STM image of eGNR on InAs (110). Scale bar is 3 nm. Scan parameters: -2 V, 8 pA. **(b)** STM image of cGNR on H:Si(100). Scale bar is 5 nm. Scan parameters: -2V, 10 pA. **(c,d)** Height profiles along the long edges of eGNR from panel (a) and cGNR from panel (b), respectively, showing the expected 1.7 nm period. **(e,f)** Height profiles across the widths of eGNR and cGNR showing an increased apparent width for the eGNR, as expected.

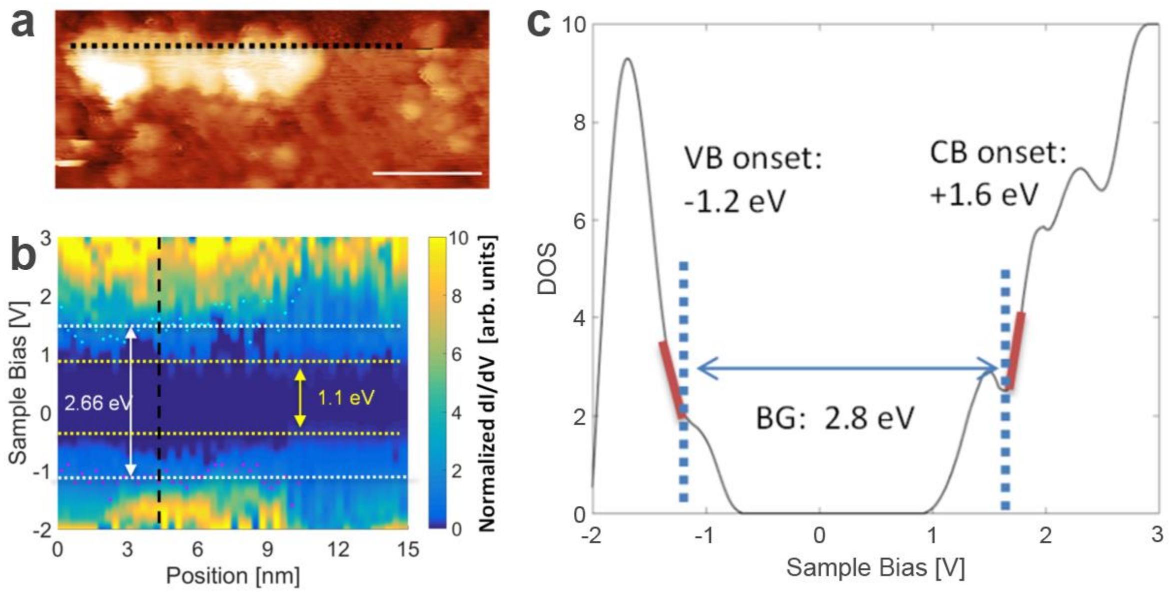

Supplementary Figure 8. Bandgap determination in eGNRs on H:Si(100). **(a)** STM topograph of eGNR on H:Si(100). Scale bar is 5 nm. Scan parameters: -3 V, 10 pA. **(b)** Normalized *dI/dV* map collected along the dashed line shown in (a) with band onsets and a 2.66 eV bandgap indicated. The valence band onsets are indicated by magenta points, and the conduction band onsets are shown in cyan. **(c)** Normalized *dI/dV* trace corresponding to the vertical dashed line in (b). Since tunneling to the substrate contributes to the measurement, the noise floor cannot be used to identify the GNR band onsets. Instead, the GNR band onsets are identified as the positions where the band edges deviate from the linear behavior. The band onsets are identified for each point along the length of the GNR, and the average positions are used to determine the bandgap.

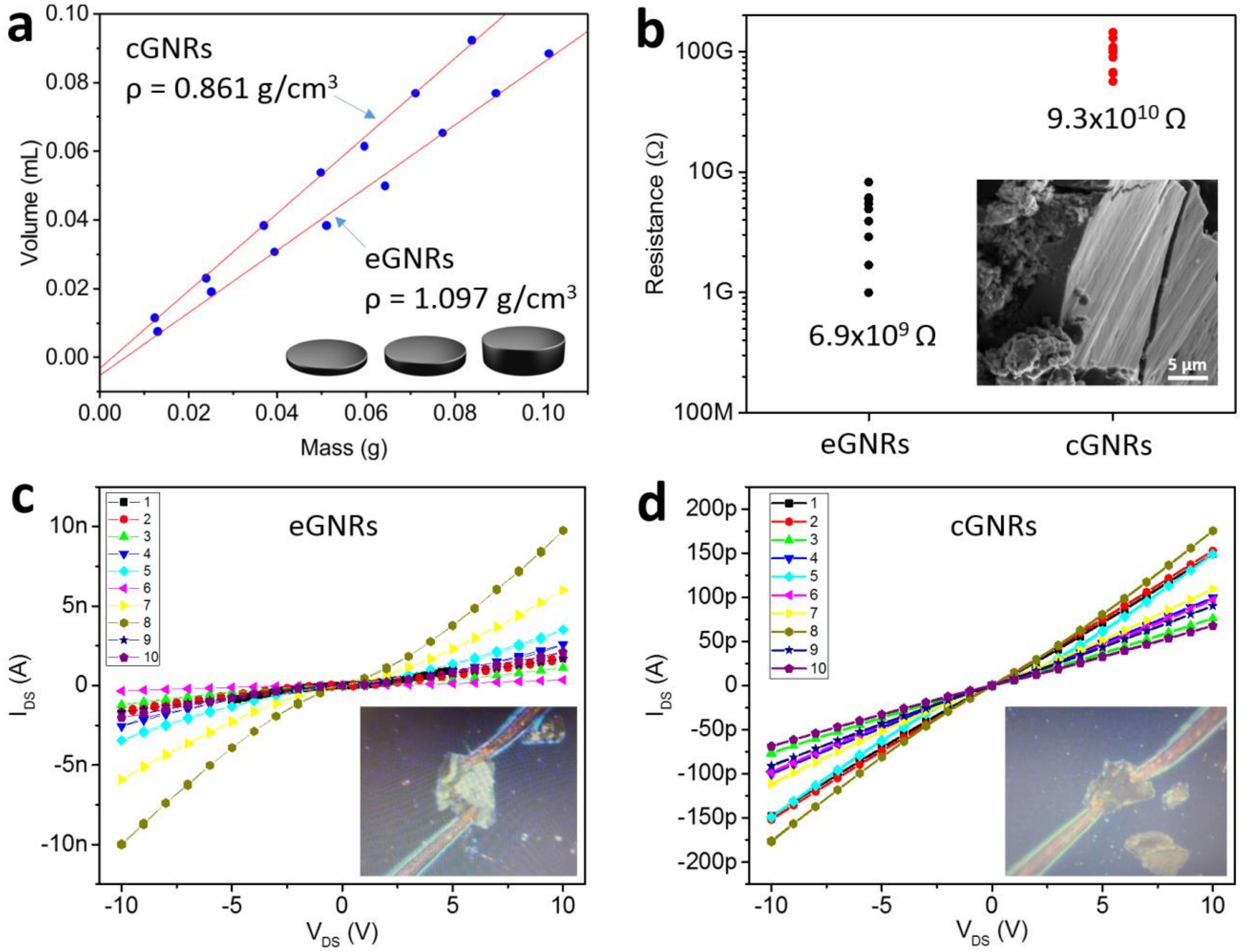

Supplementary Figure 9. (a) Density measurements of pressed pellets of eGNRs and cGNRs. **(b)** Electrical resistances of eGNR and cGNR particles. SEM image of a typical particle of eGNRs is shown in the inset. The data were extracted from I_{DS}-V_{DS} dependencies presented in panels (c,d). **(c)** I_{DS}-V_{DS} dependencies for 10 eGNR particles. Optical photograph of an eGNR particle contacted with two W tips is shown in the inset. **(d)** I_{DS}-V_{DS} dependencies for 10 cGNR particles. Optical photograph of a cGNR particle contacted with two W tips is shown in the inset.

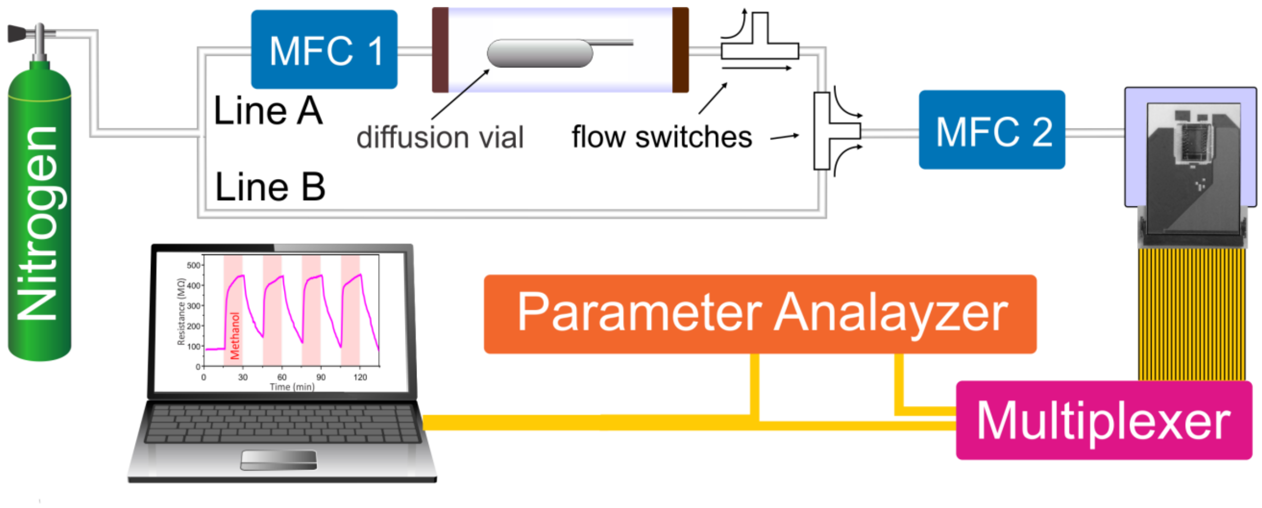

Supplementary Figure 10. Experimental setup for sensor measurements (MFC – mass flow controller). See Supplementary Note 5 for details.

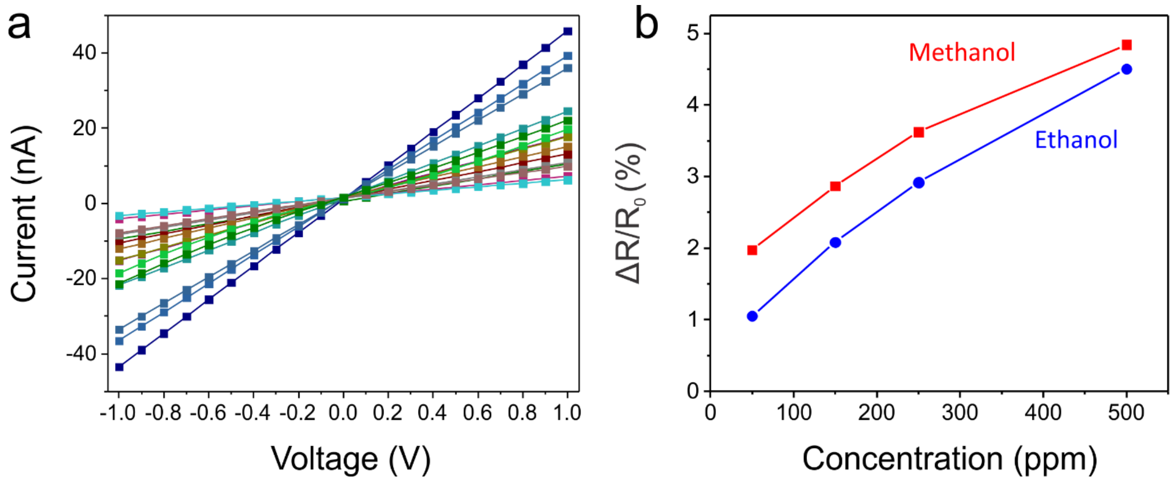

Supplementary Figure 11. (a) Current-voltage (*I*-*V*) curves for 15 eGNR segments. eGNR devices exhibit linear *I*-*V* curves, suggesting Ohmic contacts between the eGNR film and Pt electrodes. **(b)** Room temperature dependences of relative resistance changes ($\Delta R/R_0$) of a representative eGNR segment on concentrations of methanol and ethanol. There is an almost linear dependence of the response of the eGNR segment on the analyte concentrations, suggesting that the channel material rather than contact resistance is responsible for the observed sensor responses.

Supplementary Note 1. Band structure calculation

The calculations for the band structures of regular chevron GNR (cGNR) and extended chevron GNR (eGNR) with periodic boundary conditions at both DFT and GW levels were performed with the VASP package.[1,2] The Perdew-Burke-Ernzerhof (PBE) exchange-correlation functional was employed.[3] The projector augmented wave (PAW) pseudopotentials were used. The Gamma-centered k-point of 4 × 1 × 1 was used for structural relaxation and subsequent band structure calculations. Before the band structures were calculated, the GNR structures were relaxed until the maximum residual forces were less than 0.01 eV/Å. Based on the ground state obtained by DFT, quasiparticle energies were calculated with the G0W0 approximation[4] implemented in VASP. To optimize the memory requirement and computational cost, the key parameters of NBANDS=512, ENCUT=400, ENCUTGW=60 and NOMEGA=32 were employed to conduct the GW simulation for both cGNR and eGNR. The parameter NBANDS indicates the number of bands included in the GW calculation. Parameters ENCUT and ENCUTGW represent the energy cutoff of plane wave basis set for ground state calculation and response function calculation, respectively. The parameter NOMEGA determines the number of frequency points for the GW calculation.

Supplementary Note 2. Synthesis of the eGNR precursor (1)

Scheme of the synthesis of the eGNR precursor **(1)** is shown in Supplementary Figure 1.

Materials

All starting materials and solvents were purchased from Sigma-Aldrich, Acros, Alfa Aesar, EMD Millipore, and other commercial suppliers and used as received without further purification.

Synthesis of 1,3-dibromo-2-iodobenzene (4)

2,6-dibromoaniline (5.0 g, 19.9 mmol) was suspended in a mixture of water (30 mL) and concentrated hydrochloric acid (15 mL) and was cooled down to 0 °C. Sodium nitrite (1.7 g, 23.9 mmol) dissolved in water (10 mL) was added dropwise to the suspension. After one hour, potassium iodide (13.2 g, 79.7 mmol) dissolved in water (30 mL) was added dropwise to the solution. The reaction was stirred for two hours at 0 °C before dichloromethane (30 mL) was added. The reaction was stirred for four hours at room temperature before it was quenched with an aqueous solution of sodium thiosulfate. The reaction was extracted with dichloromethane, dried over anhydrous magnesium sulfate, and evaporated. Purification by silica gel column chromatography (eluent: hexane) gave the title compound as a white solid (5.42 g, 75.2 % yield): ^{1}H NMR (700 MHz, $CDCl_3$): δ = 7.54 (d, 2 H), 7.06 (t, 1 H); ^{13}C NMR (175 MHz, $CDCl_3$): δ = 131.4, 131.2, 130.4, 109.5.

Synthesis of 3'-bromo-1,1':2',1''-terphenyl (5)

Solvent system of toluene (60 mL) and water (6 mL) was degassed by nitrogen bubbling for fifteen minutes. 1,3-dibromo-2-iodobenzene **(4)** (5.42 g, 15.0 mmol), phenylboronic acid (4.02 g, 33.0 mmol), palladium(II) acetate (0.168 g, 0.75 mmol), triphenylphosphine (0.39 g, 1.50 mmol), and potassium carbonate (8.29 g, 60.0 mmol) were added sequentially. The reaction was heated to reflux and stirred under nitrogen for sixteen hours. After the reaction was allowed to cool to room

temperature, it was extracted three times with dichloromethane, dried over anhydrous magnesium sulfate, and evaporated. Purification by silica gel column chromatography (eluent: hexane) gave the title compound as a white solid (3.62 g, 78.0 % yield): ^{1}H NMR (700 MHz, $CDCl_3$): δ = 7.74 (d, 1 H), 7.42 (d, 1 H), 7.32 – 7.26 (m, 4 H), 7.18 – 7.15 (m, 5 H), 7.10 (d, 2 H); ^{13}C NMR (175 MHz, $CDCl_3$): δ = 143.7, 141.2, 141.1, 140.2, 131.9, 130.7, 129.7, 129.4, 128.8, 127.8, 127.7, 127.2, 126.7, 124.7.

Synthesis of 3'-iodo-1,1':2',1''-terphenyl (6)

Due to low reactivity towards Sonogashira coupling, a halogen exchange was performed. 3'-bromo-1,1':2',1"-terphenyl **(5)** (2.0 g, 6.47 mmol) was dissolved in anhydrous tetrahydrofuran (20 mL) and cooled to -78 °C. n-Butyllithium (2.5 M in hexanes) (3.10 mL, 7.76 mmol) was added dropwise. The reaction was stirred at -78 °C for two hours and then stirred at room temperature overnight. After cooling the reaction down to -78 °C, iodine (2.46 g, 9.71 mmol) dissolved in anhydrous tetrahydrofuran (15 mL) was added dropwise. The reaction was stirred at -78 °C for two hours and four hours at room temperature before it was quenched by addition of aqueous sodium thiosulfate. The reaction was extracted with dichloromethane, washed with water, dried over anhydrous magnesium sulfate, and evaporated. Purification by silica gel column chromatography (eluent: hexane) gave a mixture of the title compound and small amount of the starting material as a white solid (1.61 g). Due to very similar R_f values in hexane, separation was not achieved. The mixture was used as-is for the next step.

Synthesis of ([1,1':2',1''-terphenyl]-3'-ylethynyl)trimethylsilane (7)

Triethylamine (25 mL) was degassed by nitrogen bubbling for fifteen minutes. 3'-iodo-1,1':2',1"-terphenyl **(6)** (1.61 g, 4.52 mmol), bis(triphenylphosphine)palladium(II) dichloride (0.159 g, 0.226 mmol), copper(I) iodide (43.0 mg, 0.226 mmol), triphenylphosphine (0.119 g, 0.452 mmol) were added sequentially. Trimethylsilylacetylene (0.960 mL, 6.78mmol) was added added last and the

reaction was stirred under nitrogen overnight. Ethyl acetate was added to the reaction, the solid was filtered, and the filtrate evaporated. Purification by silica gel column chromatography (eluent: 10 % dichloromethane/hexane) gave the title compound as a white solid (1.32 g, 89.4 % yield): ^{1}H NMR (400 MHz, $CDCl_3$): δ = 7.60 (dd, 1 H), 7.42 – 7.35 (m, 2 H), 7.21 – 7.17 (m, 8 H), 7.11 – 7.08 (m, 2 H); ^{13}C NMR (100 MHz, $CDCl_3$): δ = 143.6, 141.6, 141.3, 139.4, 131.7, 130.9, 130.7, 129.9, 127.8, 127.4, 127.2, 126.7, 126.6, 123.6, 104.9, 97.9, 0.05.

Synthesis of 3'-ethynyl-1,1':2',1''-terphenyl (8)

([1,1':2',1''-terphenyl]-3'-ylethynyl)trimethylsilane **(7)** (1.32 g, 4.04 mmol) was dissolved in methanol (25 mL). Potassium carbonate (1.12 g, 8.09 mmol) was added, and the reaction was stirred at room temperature and monitored by TLC. After three hours, the reaction was extracted with dichloromethane, washed with water, dried over anhydrous magnesium sulfate, and evaporated to give the title compound as an off-white solid (0.983 g, 95.6 % yield).

5,10-dibromo-1,3-diphenyl-2H-cyclopenta[l]phenanthren-2-one (9)

The synthesis of this compound was reported in our previous paper.[5]

Synthesis of 2-([1,1':2',1''-terphenyl]-3'-yl)-6,11-dibromo-1,4-diphenyltriphenylene (1)

To a mixture of 5,10-dibromo-1,3-diphenyl-2H-cyclopenta[l]phenanthren-2-one **(9)** (2.51 g, 4.64 mmol) and 3'-ethynyl-1,1':2',1''-terphenyl **(8)** (0.983 g, 3.87 mmol) was added diphenyl ether (2 mL). The reaction was heated to reflux and stirred overnight. Reaction progress was monitored by TLC. After the reaction was allowed to cool to room temperature, it was diluted with dichloromethane and dried under vacuum. Purification by silica gel column chromatography (eluent: 5 % ethyl acetate/hexane) gave the title compound as an off-white solid (1.88 g, 63.4 % yield): ^{1}H NMR (700 MHz, $CDCl_3$): δ = 8.17 (dd, 2 H), 7.79 (d, 1 H), 7.69 (d, 1 H), 7.54 – 7.39 (m, 12 H), 7.29 – 7.19 (m, 4 H), 7.13 – 7.09 (m, 4 H), 7.04 – 6.89 (m 4H), see Supplementary Figure 2; ^{13}C NMR (175 MHz,

$CDCl_3$): δ = 143.4, 141.9, 141.6, 141.1, 140.6, 140.3, 139.3, 138.3, 137.7, 137.1, 134.5, 134.4, 133.1, 132.5, 132.4, 131.6, 131.0,130.9, 129.8, 129.7, 129.6, 129.4, 129.3, 129.2, 128.9, 128.5, 127.7, 127.5, 126.9, 126.6, 126.0, 125.7, 124.5,124.4, 119.9, 119.7, see Supplementary Figure 3. Due to the rotation barrier, not all carbons were observed. The attempt of high-temperature NMR in DMSO was unsuccessful.

Supplementary Note 3. DFT calculations of the Raman spectrum

The calculations of the Raman spectrum were performed using the plane-wave density functional theory code Quantum ESPRESSO.[6] We used the exchange–correlation functional of Perdew, Burke, and Ernzerhof (PBE)[3] together with norm-conserving pseudopotentials developed by the Rappe Group.[7] The smallest repetition unit of the studied nanoribbon was placed in an orthorhombic cell, propagating along the X-direction and maintaining separation of at least 20 bohr (approx. 10.6 Å) on the vacuum sides (Y and Z). A large cutoff of 100 Ry for the electronic wavefunction and a tight convergence threshold of 10^{-12} for self-consistency were used throughout the calculations. The phonon frequencies and Raman activities were computed at the Γ point of the Brillouin zone using the density-functional perturbation-theory for phonons[8] and second-order response for Raman activities,[9] for a structure previously relaxed to the level of very small forces ($<6.5\times10^{-5}$ Ry per bohr). The visualization of the displacement pattern for the RBM-like Raman mode of eGNR (Figure 2d in the main text) was prepared using XCrysDen[10] operating under Silicon Graphics IRIX 6.5.

For comparison with the experimental Raman spectrum the calculated Raman activities for the back scattering geometry S_j were converted into differential Raman scattering cross sections using the following equation:[11]

$$\frac{d\sigma_j}{d\Omega} \approx \frac{(\nu_0 - \nu_j)^4}{\nu_j} \frac{S_j}{1 - \exp\left(-\frac{hc\nu_j}{kT}\right)},$$

where ν_0 and ν_j are the frequencies (wavenumbers) of the excitation line (18797 cm^{-1} corresponding to 532 nm) and the j-th normal mode, respectively; h, c and k are the universal constants. The temperature T was chosen to be 300K.

Supplementary Note 4. Comparison of bulk properties of eGNRs and cGNRs

We pressed a series of pellet from eGNR and cGNR powders and plotted their volumes versus masses (Supplementary Figure 9a). The data were fitted with linear dependencies and from their slopes we determined densities of pressed eGNR and cGNR materials. We found that eGNRs form denser pellets ($\rho = 1.1$ g/cm^3) than cGNRs ($\rho = 0.86$ g/cm^3), which suggests their stronger aggregation. Scanning electron microscopy (SEM) analysis of as-synthesized eGNR powder revealed the presence of dense particles of nanoribbons with sizes up several tens of μm, see the inset in Supplementary Figure 9b and Supplementary Figure 6. The Raman spectra recorded from these particles were consistent with the data shown in the main text in Figure 2b,c.

While electrical characterization of individual GNRs remains a great challenge, in this work we tested electronic properties of macroscopic assemblies of eGNRs and cGNRs. The preparation and analysis of self-assembled eGNR and cGNR films is described in the main text. We also measured resistances of GNR particles similar to the one shown in the inset in Supplementary Figure 9b; the results of these measurements are summarized in Supplementary Figure 9b-d. The electrical measurements were performed inside a Lake Shore TTPX cryogenic probe station at the base pressure of 2×10^{-6} Torr. The eGNR and cGNR particles were deposited on Si substrates covered with 300-nm-thick SiO_2. Particles with similar sizes (100-150 μm across) were directly contacted with W tips of a probe station, which served as source (S) and drain (D) electrodes in these measurements; optical photographs of particles of eGNRs and cGNRs contacted with W tips inside the probe station are shown in the insets in Supplementary Figures 9c and 9d, respectively. The drain-source current (I_{DS}) – drain-source voltage (V_{DS}) dependencies were measured using an Agilent 4155C semiconductor parameter analyzer that was linked to a computer through 82357B USB/GPIB interface and controlled using a National Instrument LabView code. While the experimental setup allowed application of gate

voltage using the p-doped Si as a bottom gate electrode, we did not observe any gate dependences of the conductivities of eGNR and cGNR particles, which is likely due to their large size.

We tested 10 particles of each type, all I_{DS}-V_{DS} dependencies for both eGNRs and cGNRs are shown in Supplementary Figures 9c and 9d, respectively. All eGNR particles were more conductive than cGNR particles. The resistance values extracted from Supplementary Figure 9c,d are summarized in Supplementary Figure 9b. On average, eGNR particles had a resistance of $6.9 \cdot 10^9$ Ω, while cGNR particles with similar sizes had an average resistance of $9.3 \cdot 10^{10}$ Ω.

It is interesting to analyze whether the lateral extension of the cGNR and the corresponding band gap reduction are consistent with the increase in electrical conductivity of eGNRs compared to cGNRs by more than an order of magnitude. According to the STS measurements, the eGNR is estimated to have a bandgap of 2.63 eV, which is smaller than the measured 2.76 eV bandgap of the parent cGNR. If we ignore the role of doping, the intrinsic carrier concentration can be estimated as

$$n_i = \sqrt{N_c N_v} e^{-Eg/_{2kT}}$$

where n_i is the intrinsic carrier concentration, N_c and N_v are the densities of states of the conduction and valence bands, respectively, Eg is bandgap, k is the Boltzmann's constant, and T is temperature. If we assume that the densities of states of the eGNR and cGNR are identical, except for a difference in bandgaps, then we can estimate the difference in intrinsic carrier concentrations. At room temperature, the 0.13 eV bandgap difference results in the increase in carrier concentrations in the eGNR versus the cGNR by a factor of 12.9. At 100 °C the intrinsic carrier concentration in eGNR would be 7.56 times that in cGNR. A similar analysis using the GW bandgaps of cGNR (3.78 eV) and eGNR (3.38 eV) shows that the carrier concentration in eGNR would be larger than that in cGNR by a factor of ~2600 at room temperature, and a factor of ~500 at 100 °C.

Supplementary Note 5. Sensor measurements of eGNRs

The setup for gas sensing measurements is shown in Supplementary Figure 10; we used the same setup for the sensor studies of other graphene materials, such as reduced graphene oxide and graphene.[12,13] Two-terminal resistance measurements of individual sensing elements in the array were performed at a constant voltage mode using an Agilent 4155C semiconductor parameter analyzer that was linked to a computer through 82357B USB/GPIB interface and controlled using a National Instruments LabView code. The parameter analyzer was connected to a Keithley 7001 switch system (multiplexer) that was sequentially reading the current from every individual sensing element in the array. The chip with an array of eGNR devices was placed into a gas exposure chamber (V ~ 2 cm^3). An analyte was put in a vial with a custom-made horizontal capillary diffusion tube (Supplementary Figure 10). If the diffusion vial is kept at a constant temperature, the concentration gradient of the analyte in and outside the vial remains constant which provides the constant driving force for a controlled release of the analyte to the flow of nitrogen. The bore diameter and diffusion path length determine the release rate for a specific analyte. The Equation (1) was used to calculate the diffusion path length:

$$L = 1.9 \cdot 10^4 \cdot T \cdot D \cdot M \cdot A \cdot \frac{\log P}{P - \rho} \cdot K \cdot \frac{1}{F \cdot C} \qquad (1),$$

where L is the length of diffusion path (cm), T is the temperature of the vapor (K), D is the diffusion coefficient (cm^2/sec) at 25˚C and 1 atm, M is the molecular weight (g/mol), A is the cross-section area of the capillary (cm^2), P is the atmospheric pressure (mm Hg), ρ is the vapor pressure of chemical at the temperature T (mm Hg), K is the molar volume constant at 25 ˚C and at 1 atm (K = 24.47/M), F is the total dilution flow (sccm), and C is the concentration, parts per million (ppm) by volume.

We used Line A to expose the eGNR devices to an analyte, and Line B to purge them with dry nitrogen (Supplementary Figure 10). Two independent mass flow controllers (MFC, Matheson Transducer, Model 8141) were used to maintain the same gas flow rates through both lines, and two flow switches were used to open and close these lines. When the eGNR devices where purged with dry nitrogen through Line B, the chamber with the diffusion vial was also continuously purged with dry nitrogen to prevent the accumulation of an analyte in the chamber.

Supplementary References

1 Kresse, G. & Furthmüller, J. Efficiency of ab-initio total energy calculations for metals and semiconductors using a plane-wave basis set. *Comput. Mater. Sci.* **6**, 15-50 (1996).

2 Kresse, G. & Furthmüller, J. Efficient iterative schemes for ab initio total-energy calculations using a plane-wave basis set. *Phys. Rev. B* **54**, 11169 (1996).

3 Perdew, J. P., Burke, K. & Ernzerhof, M. Generalized Gradient Approximation Made Simple. *Phys. Rev. Lett.* **77**, 3865-3868 (1996).

4 Shishkin, M. & Kresse, G. Self-consistent G W calculations for semiconductors and insulators. *Phys. Rev. B* **75**, 235102 (2007).

5 Vo, T. H., Shekhirev, M., Kunkel, D. A., Morton, M. D., Berglund, E., Kong, L. M., Wilson, P. M., Dowben, P. A., Enders, A. & Sinitskii, A. Large-Scale Solution Synthesis of Narrow Graphene Nanoribbons. *Nat. Commun.* **5**, 3189 (2014).

6 Giannozzi, P., Baroni, S., Bonini, N., Calandra, M., Car, R., Cavazzoni, C., Ceresoli, D., Chiarotti, G. L., Cococcioni, M., Dabo, I., Dal Corso, A., de Gironcoli, S., Fabris, S., Fratesi, G., Gebauer, R., Gerstmann, U., Gougoussis, C., Kokalj, A., Lazzeri, M., Martin-Samos, L., Marzari, N., Mauri, F., Mazzarello, R., Paolini, S., Pasquarello, A., Paulatto, L., Sbraccia, C., Scandolo, S., Sclauzero, G., Seitsonen, A. P., Smogunov, A., Umari, P. & Wentzcovitch, R. M. QUANTUM ESPRESSO: a modular and open-source software project for quantum simulations of materials. *J. Phys.-Condes. Matter* **21**, 395502 (2009).

7 Pseudopotentials in the Fritz-Haber-Institute (FHI) format available from: http://www.sas.upenn.edu/rappegroup/research/pseudo-potential-gga.html.

8 Baroni, S., de Gironcoli, S., Dal Corso, A. & Giannozzi, P. Phonons and related crystal properties from density-functional perturbation theory. *Rev. Mod. Phys.* **73**, 515-562 (2001).

9 Lazzeri, M. & Mauri, F. First-Principles Calculation of Vibrational Raman Spectra in Large Systems: Signature of Small Rings in Crystalline SiO_2. *Phys. Rev. Lett.* **90**, 036401 (2003).

10 Kokalj, A. Computer graphics and graphical user interfaces as tools in simulations of matter at the atomic scale. *Comput. Mater. Sci.* **28**, 155-168 (2003).

11 Murphy, W. F., Holzer, W. & Bernstein, H. J. Gas Phase Raman Intensities: A Review of “Pre-Laser” Data. *Appl. Spectrosc.* **23**, 211-218 (1969).

12 Lipatov, A., Varezhnikov, A., Wilson, P., Sysoev, V., Kolmakov, A. & Sinitskii, A. Highly selective gas sensor arrays based on thermally reduced graphene oxide. *Nanoscale* **5**, 5426-5434 (2013).

13 Lipatov, A., Varezhnikov, A., Augustin, M., Bruns, M., Sommer, M., Sysoev, V., Kolmakov, A. & Sinitskii, A. Intrinsic device-to-device variation in graphene field-effect transistors on a Si/SiO_2 substrate as a platform for discriminative gas sensing. *Appl. Phys. Lett.* **104**, 013114 (2014).
